# Venereal and Common Warts

**Published:** 1883-06

**Authors:** 


### Venereal and Common Warts.

Unna (*Boston Medical and Surgical Journal*) recommends for the treatment of condylomata acuminata and ordinary warts the continuous application of unguent hydrarg. containing five per cent. of arsenic. In the case of a young girl upon whose hands were a hundred or more warts, the unbroken application for three weeks of a plaster containing each 0.2 square metre, 10.00 grammes of arsenic and five grammes of mercury, caused entire disappearance of the disease without any irritation of the normal skin. Cure was effected not by reason of necrosis and destruction of the warts, as after the use of caustics, but by resorption, as in cases of spontaneous cure.—*Chic. Med. Review.*

